# Supplementary material for: Austalide K from the Fungus Penicillium rudallense Prevents LPS-Induced Bone Loss in Mice by Inhibiting Osteoclast Differentiation and Promoting Osteoblast Differentiation
Source: Int J Mol Sci. 2021 May 23;22(11):5493. doi: 10.3390/ijms22115493 (PMC8197085; doi:10.3390/ijms22115493)

Supporting Information for

Austalide K, from the Fungus *Penicillium rudallense* Prevents LPS-Induced Bone Loss in Mice by Inhibiting Osteoclast Differentiation and Promoting Osteoblast Differentiation

Kwang-Jin Kim[1,†], Jusung Lee[2,†], Weihong Wang[2,4,†], Yongjin Lee[1], Eunseok Oh[2], Kyu-Hyung Park[2], Chanyoon Park[3], Gee-Eun Woo[2], Young-Jin Son[1,] and Heonjoong Kang[2,3,4,*]*

1 Department of Pharmacy, Sunchon National University, 315 Maegok-dong, Suncheon, Jeollanam-do 57922, Korea; mastiffk@naver.com (K.-J.K.), yojilee@gmail.com (Y.L.)

2 Laboratory of Marine Drugs, School of Earth and Environmental Sciences, Seoul National University, NS-80, Seoul 08826, Korea; leejusung@snu.ac.kr (J.L.); pharmacy2007@naver.com (W.W.); dmstjr0130@snu.ac.kr (E.O.); grekhp@snu.ac.kr (K.-H.P.); tddok7953@naver.com (G.-E.W.)

3 Interdisciplinary Graduate Program in Genetic Engineering, Seoul National University, NS-80, Seoul 08826, Korea; chanyoon0126@snu.ac.kr (C.P.)

4 Research Institute of Oceanography, Seoul National University, NS-80, Seoul 08826, Korea

* Co-correspondence: sony@scnu.ac.kr; Tel.: 82-61-750-3755, hjkang@snu.ac.kr; Tel.: 82-2-880-5730

† These authors contributed equally to this work.

Structural elucidation of austalide K

The molecular formula of austalide K was determined to be $C_{25}H_{32}O_5$ based on analysis of its molecular ion peak in the HRFABMS spectrum and ^{13}C NMR data. The IR spectrum of austalide K showed characteristic absorption at 1748 cm^{-1} and 1700 cm^{-1}, indicating presence of an ester carbonyl and a ketone group. The UV maxima (λ_{max} 216 and 267 nm) indicated the existence of a phthalide moiety in the molecule.

The ^{1}H and ^{13}C NMR spectra exhibited signals of pentacyclic backbone in austal ide K. Six singlet methyls including one aromatic methyl group (H25 : δ 2.04), one methoxy group (H11 : δ 4.11), four aliphatic groups (H19 : δ 1.19, H20 : δ 1.12, H21 : δ1.03, H24 : δ 0.72) were observed. Additionally, six methylenes (C15 : δ 29.9, C16 : δ 38.5, C18 : δ 34.2, C22 : δ 19.2, C23 : δ 18.4) with one oxygenated (C10 : δ 68.4) were determined. Two methines (C12 : δ 54.3, 14 : 47.2) and nine non protonated carbons were observed (Supplementary Table 1). The ^{13}C NMR spectrum showed signals at δ 216.8 (C 1) and 169.6 (C-2), which were assigned to ketone carbonyl an d ester carboxyl carbons, respectively. The HMBC correlations established the structure of the rings and the linkage of the pentacyclic moiety (Fig 1)

Table S1 ^1H (700 MHz) and ^{13}C NMR (175 MHz) data of austalide K in $CDCl_3$ (*J* in Hz)

**No.**	**δ_C**	δ_H, mult[b] (*J* in Hz)
1	216.8	
2	169.6	
3	158.8	
4	155.5	
5	145.7	
6	115.4	
7	114.6	
8	107.5	
9	76.3	
10	68.4	5.12 (s)
11	62.1	4.11 (s)
12	54.3	1.48 (d, m)
13	47.4	
14	47.2	1.49 (m)
15	39.9	2.29 (dt, 14.1, 2.80)
		1.66 (td, 14.1, 4.3)
16	38.5	2.10 (ddd, 11.7, 7.0, 3.8)
		1.51 (m)
17	37.8	
18	34.2	2.53 (ddd, 16.1, 11.7, 7.0)
		2.41 (ddd, 16.1, 6.4, 3.8)
19	27.2	1.19 (s)
20	26.8	1.12 (s)
21	21.8	1.03 (s)
22	19.2	1.81 (qd, 13.0, 3.5)
		1.52 (m)
23	18.4	2.93 (d, 18.5)
		2.81 (dd, 18.5, 8.1)
24	14.3	0.72 (s)
25	10.8	2.04 (s)

Figure S1 ^{1}H NMR spectrum (700 MHz, $CDCl_3$) of austalide K

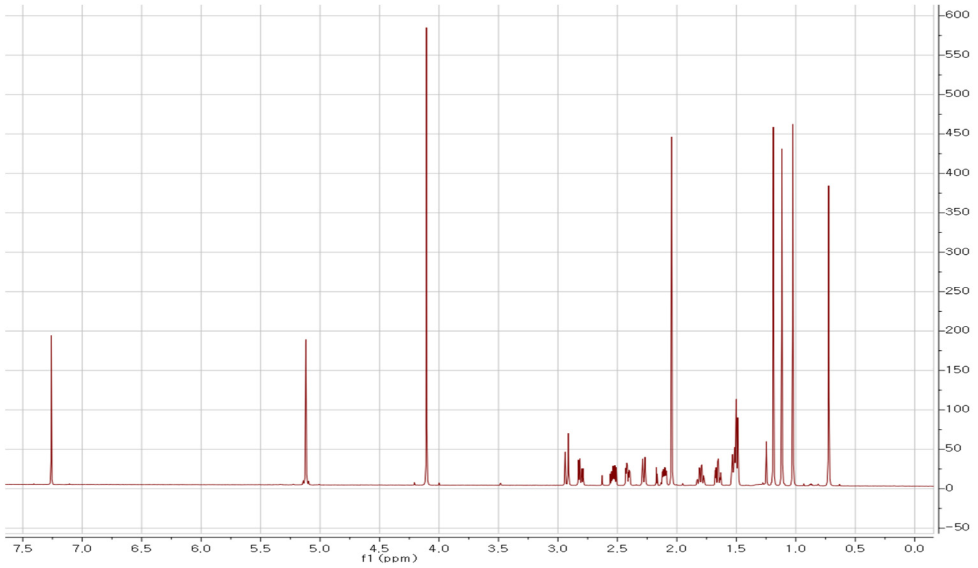

Figure S2 ^{13}C NMR spectrum (175 MHz, $CDCl_3$) of austalide K

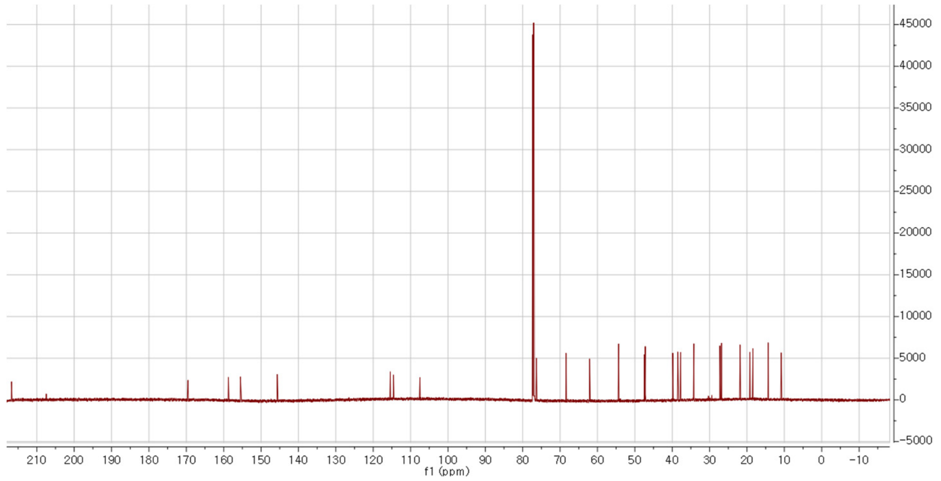

Figure S3 HSQC spectrum (700 MHz, $CDCl_3$) of austalide K

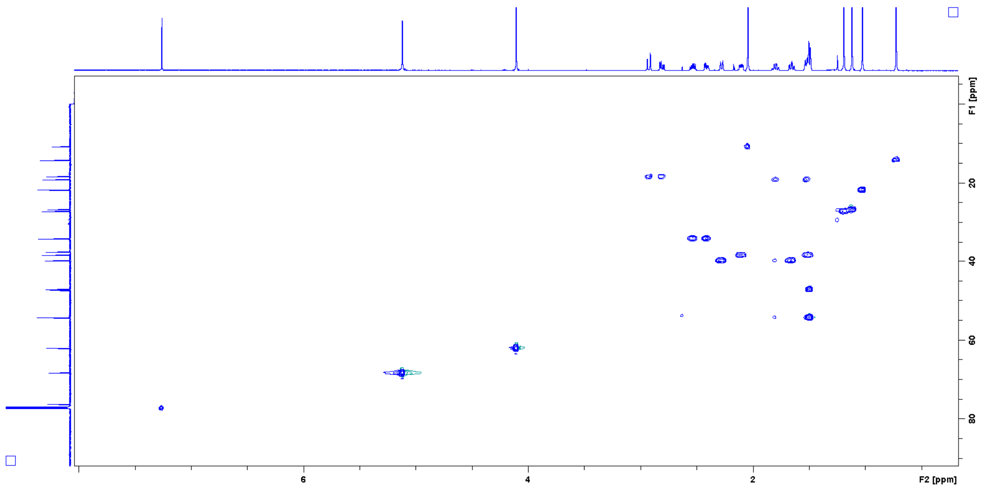

Figure S4 HMBC spectrum (700 MHz, $CDCl_3$) of austalide K

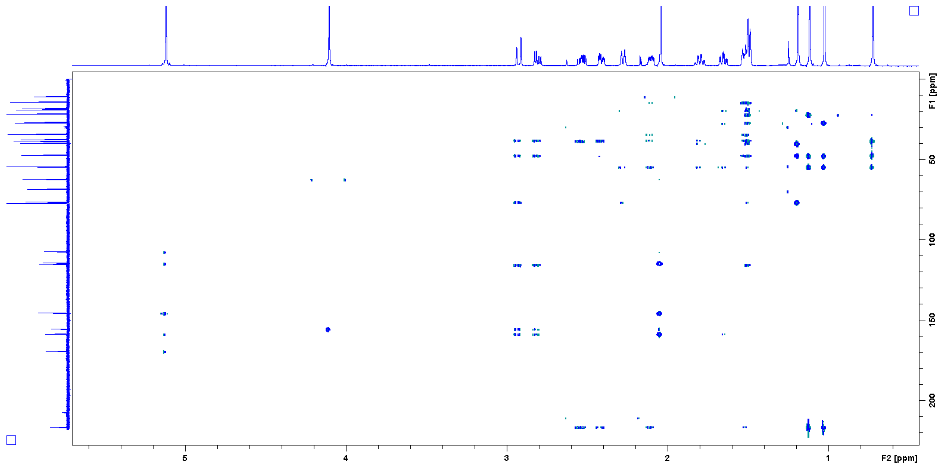

Figure S5 COSY spectrum (175 MHz, CDCl3) of austalide K

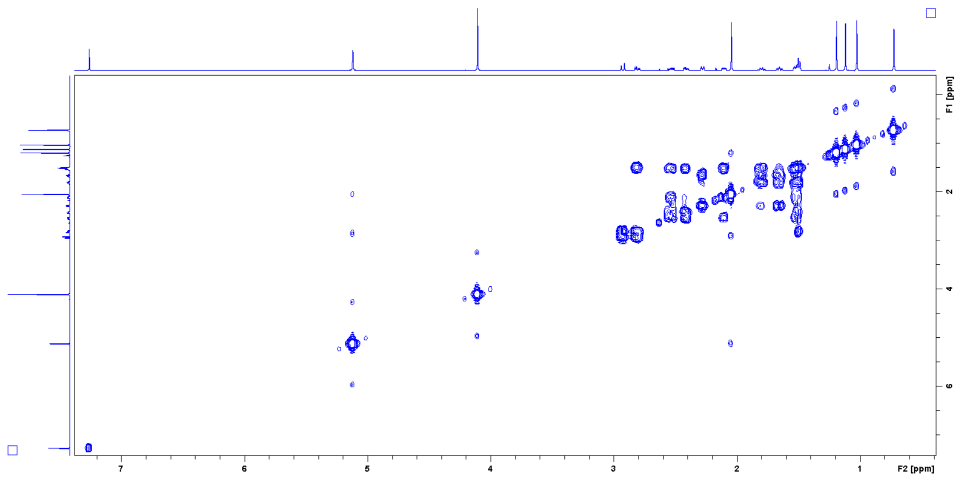

Figure S6 ROESY spectrum (175 MHz, CDCl3) of austalide K

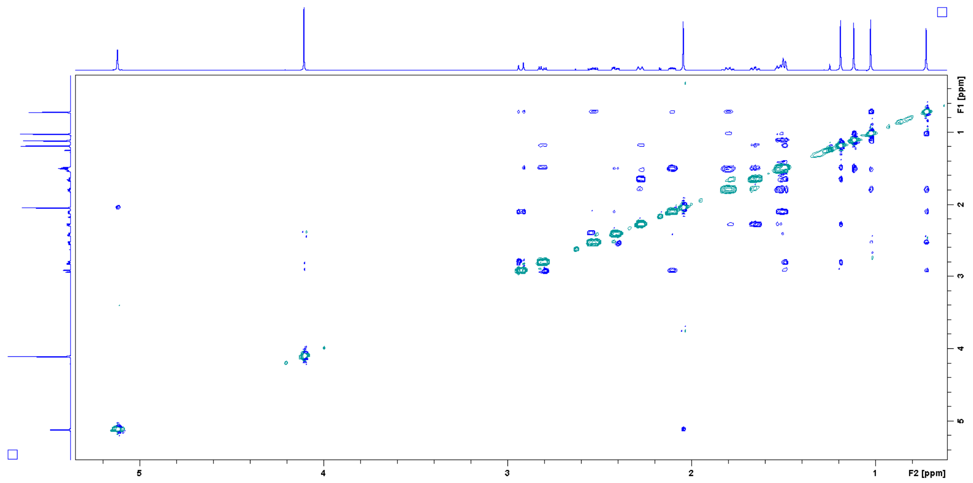

Figure S7 The expression level of NFATc1 was measured by using western blot analysis.

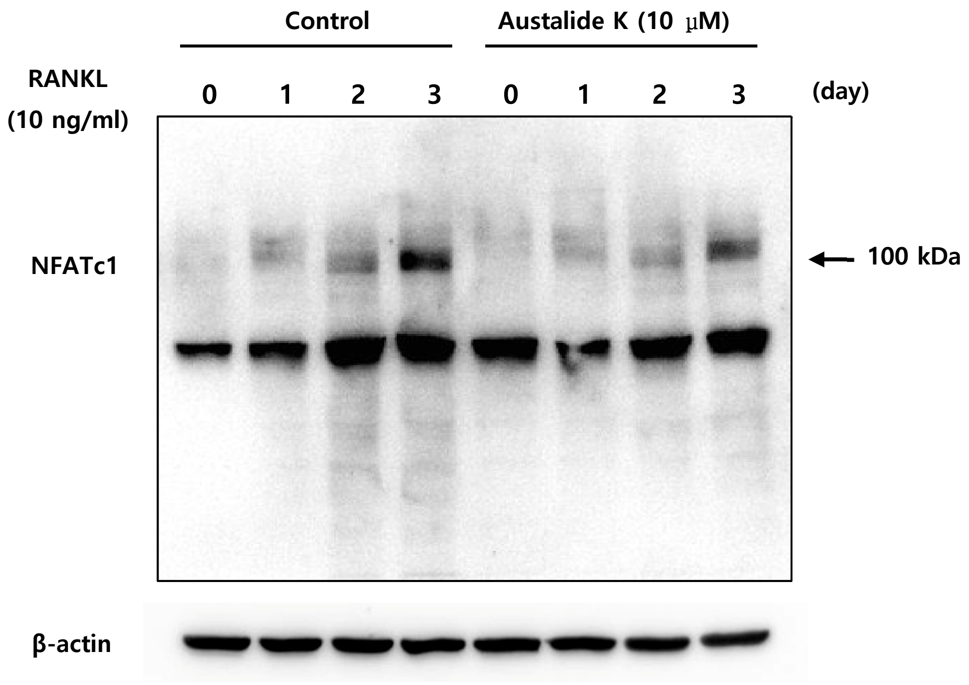

Figure S8 Survival rate (%) of RANKL-induced BMM with treatment of austalide K.

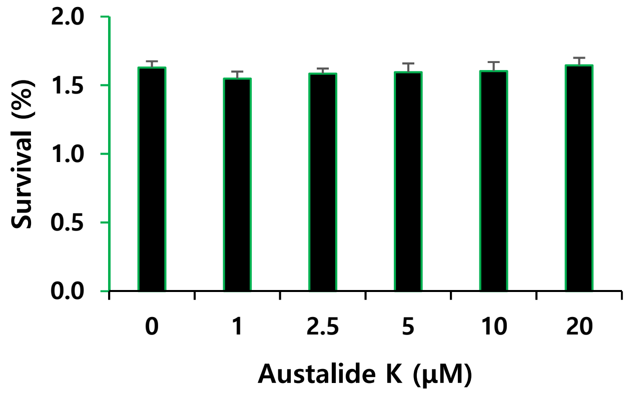

Supplement: Supplementary file 1 [file ijms-22-05493-s001.zip › ijms-1200016-supplementary.pdf]
